# Cortical midfrontal theta dynamics following foot strike may index response adaptation during reactive stepping

**DOI:** 10.1038/s41598-022-22755-3

**Published:** 2022-10-22

**Authors:** Mitchel Stokkermans, Wouter Staring, Michael X. Cohen, Teodoro Solis-Escalante, Vivian Weerdesteyn

**Affiliations:** 1 Donders Institute for Brain, Cognition and Behaviour, Radboud University Medical Center, Reinier Postlaan 4, 6525 GC Nijmegen, The Netherlands; 2 Department of Synchronisation in Neural Systems, Donders Institute for Brain Cognition and Behavior, Kappitelweg 29, 6525 EN Nijmegen, The Netherlands; 3 Sint Maartenskliniek Research, Hengstdal 3, 6574 NA Nijmegen, The Netherlands

**Keywords:** Neuroscience, Cognitive neuroscience, Motor control

## Abstract

Reactive balance recovery often requires stepping responses to regain postural stability following a sudden change in posture. The monitoring of postural stability has been linked to neuroelectrical markers such as the N1 potential and midfrontal theta frequency dynamics. Here, we investigated the role of cortical midfrontal theta dynamics during balance monitoring following foot landing of a reactive stepping response to recover from whole-body balance perturbations. We hypothesized that midfrontal theta dynamics reflect the engagement of a behavioral monitoring system, and therefore that theta would increase time-locked to the moment of foot strike after a stepping response, coinciding with a re-assessment of postural balance to determine if an additional step is necessary. We recorded high-density EEG and kinematic data of 15 healthy young participants while they stood on a platform that delivered multi-directional balance perturbations. Participants were instructed to recover balance with a single step utilizing either their left or right leg (in separate blocks). We used targeted spatial filtering (generalized eigen decomposition) in combination with time–frequency analysis of the EEG data to investigate whether theta dynamics increase following foot strike event. In line with our hypothesis, the results indicate that the foot strike event elicits a midfrontal theta power increase, though only for backward stepping. Counter to our expectations, however, this theta power increase was positively correlated with the margin of stability at foot strike, suggesting a different role of foot strike related theta from monitoring stability. Post-hoc analysis suggests that midfrontal theta dynamics following foot landing may instead facilitate adaptation of stability margins at subsequent stepping responses. We speculate that increase of theta power following foot strikes was not related to stability monitoring but instead may indicate cortical dynamics related to performance monitoring of the balance response.

## Introduction

On a daily basis, we experience challenges to our postural stability, either from errors when engaging in voluntary movements (e.g. misjudging the height of a step down) or from external perturbations (e.g. being pushed). Such instances often require a corrective step to maintain balance. This generally involves little difficulties for young healthy individuals but can be challenging or even dangerous in individuals with impaired balance or in elderly^1,2^.

Although balance control was traditionally considered a subcortical function, it has become evident that the cerebral cortex is also involved. In particular, two cortical signals (measured through Electroencephalogram; EEG) have been consistently observed shortly after an unexpected balance perturbation: the N1 event-related potential, and power modulations of theta-band (3–8 Hz) activity^3,4^. The roles of N1 and theta power increase have been linked to sensory integration and facilitating cognitive processes underlying postural control (e.g. balance monitoring), supporting evidence for these roles comes from studies that measured continuous balance performance and found that a less stable posture correlated with stronger theta power^5,6^. Additionally, theta power scales with both balance perturbation intensity and with stepping or feet-in-place (i.e. to overcome a balance perturbation without making a step) response outcomes, suggesting that theta signals the loss of stability and predicts the necessity of a step^7^. Furthermore, we recently showed that manipulating the leaning posture prior to random perturbation intensities resulted in theta power modulations that scaled with postural threat (i.e. greater postural threat caused theta dynamics to rise faster over smaller perturbation intensities^8^. Altogether these results show that theta dynamics are intimately involved in balance monitoring.

In light of this supporting evidence for a role in balance monitoring shortly after an unexpected perturbation, we reasoned that a theta power increase may also be elicited at the moment when the stepping foot strikes the ground, based on the idea that the new postural stability after the recovery step is not guaranteed and may require a re-assessment of the new postural state.

In this study, we investigated foot strike related theta power dynamics in reactive stepping trials following unexpected balance perturbations in forward and backward directions. We hypothesized that the foot strike event is followed by a midfrontal theta power increase relative to baseline theta power and distinguishable from the initial perturbation-induced theta power increase. In addition, we expected that these theta power dynamics represent a balance monitoring process of the new postural state after foot strike. We therefore expected that stronger theta dynamics scale with lower margins of stability at foot contact, as a commonly used measure of dynamic stability that captures the relationship between center of mass and the edge of the base of support at foot strike. We also studied whether theta power dynamics may differ between forward and backward reactive stepping responses, because there are behavioral differences between perturbation directions. Previous studies showed that there are evident biomechanical differences in the forward and backward reactive stepping response^9,10^ and visual control of the step also differs between these directions. In addition, backward reactive steps occur faster than forward reactive steps^7,11^ and at lower stepping thresholds (i.e. lower perturbation intensities^12,13^). Given the relatively greater challenge involved in backward as compared to forward steps, we expected theta dynamics to be stronger in the backward direction.

## Materials and methods

### Participants

Fifteen young healthy adults (6 female; mean age 24 years, sd 2 years) participated in this study. None of the participants had a previous history of neurological or musculoskeletal conditions or other impairments that could affect balance. They provided written informed consent before participating in the experiment according to the experimental procedures approved by the Research Ethics Committee of the Radboud University Medical Center (Nijmegen, The Netherlands; Dossier NL67690.091.18). The experiments were conducted in line with the Declaration of Helsinki.

### Experimental paradigm

**Figure 1:**
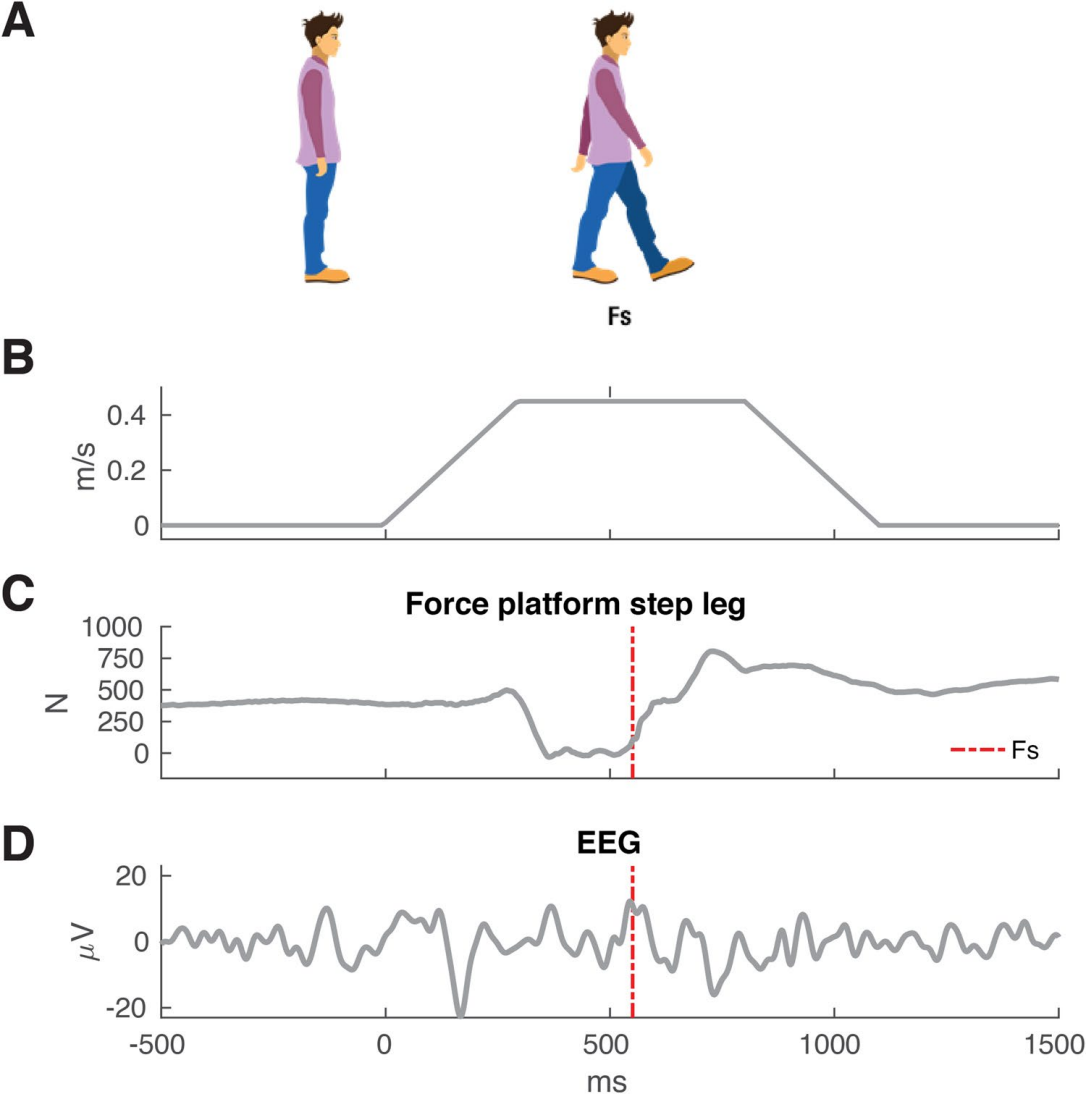
Experimental data alignment. (**A**) The participant’s response to a forward perturbation (i.e. backward platform translation). Foot strikes (Fs) were identified from the force plate and marker data. (**B**) The perturbation profile used in the experiment expressed in velocity over time. The total perturbation duration was 1.1 s. (**C**) Vertical forces measured underneath the stepping leg, with the vertical dashed line indicating the instant of foot strike of the stepping leg (Fs). (**D**) Trial-specific Fs time stamps were used to time- lock EEG data for data analysis. For illustration purposes, EEG data shown in this figure is 10 Hz low-pass filtered single-trial EEG data time-locked to perturbation onset.

The Radboud Falls Simulator was used to deliver balance perturbations and to assess reactive stepping responses^14^. This movable platform is equipped with embedded dual force plates, (60 by 180 cm each, AMTI Custom 6 axis composite force platform, USA) and a 3D motion capture system (Vicon Motion Systems, Oxford, UK). Translations of the movable platform elicit a reactive stepping response in the opposite direction of the platform translation (i.e. backward platform translations result in forward stepping). Henceforth, we will refer to stepping direction when mentioning perturbation direction. Participants were instructed to stand upright with their feet 5 cm apart (marked on the platform) and to recover their balance with a single step. Prior to each block of perturbations, participants were instructed to use either their right or their left leg for stepping. Perturbations were given in five different directions for each leg (backward, diagonal backward, sideways, diagonal forward and forward). The perturbation profile consisted of an acceleration of 1.5 m/s^2^ for 300 ms followed by a constant velocity phase of 500 ms and a deceleration phase of 300 ms (see Fig. 1). During the experiment, participants wore a safety harness that was attached to a sliding rail on the ceiling.

Participants performed four trial blocks with each leg. Each block consisted of 25 perturbations, with five repetitions in each perturbation direction in a random order. In this study we restricted our analyses to the forward and backward platform perturbations, merging the responses for the left and right-leg stepping trials (i.e. 40 trials in forward and 40 trials in backward directions).

### Data collection

We recorded high-density EEG using a cap with 126 Ag–AgCl electrodes (WaveGuard, ANT Neuro, The Netherlands). The electrodes were fixed in the cap and distributed across the scalp according to the five percent electrode system (Oostenveld and Praamstra, 2001). The EEG was referenced to the common average during acquisition. The ground electrode was placed on the left mastoid. A biosignal amplifier (REFA System, TMSi, The Netherlands) recorded the EEG at 2048 Hz without any filters, except for a hardware low-pass filter at 552 Hz. To monitor physiological activity that could present artifacts in the EEG, we also recorded electrical activity of the left eye in the vertical and horizontal direction (electrooculogram, EOG) using adhesive Ag–AgCl electrodes. The EOG was recorded from electrodes placed slightly under the left eye (vertical eye movement) and at the outer canthus of the left eye (horizontal eye movement).

Body movements were recorded using an 8-camera 3D motion analysis system (Vicon motion systems, United Kingdom) at a sample rate of 100 Hz. For this purpose, a total of 23 reflective markers (PlugInGait Full-body AI model excluding the head and arm markers; Vicon Nexus software 2.7.1) were attached to anatomical landmarks on the participants’ body.

### Processing and analysis

Force plate data were used to identify the moment of foot strike during a trial (see Fig. 1). The threshold for foot strike identification was set at a participant-specific level of 10% bodyweight loading on one leg. Foot strikes were visually verified by a subsequent lack of foot marker (ankle, heel or toe) movement following an identified foot strike. We discarded trials with foot strikes occurring beyond the constant velocity phase of the perturbation (i.e. 0.8 s post perturbation onset) for further analysis to avoid potential effects of platform deceleration on the balance recovery response^15^.

Markers were labeled and reconstructed in a batch preprocessing pipeline. Occluded markers on the hip or trunk segment were gap-filled with a rigid body fill algorithm when at least three other markers of the body segment were visible. Other missing markers were gap filled using the Vicon Woltring filter, but only if they did not overlap foot strike event (± 0.5 s) and lasted a maximum of 50 frames. Marker data were imported to MATLAB and 10 Hz low-pass filtered (5th order Butterworth IIR filters, zero-phase shift). The foot strike events were merged into the EEG datasets.

#### Margin of stability

Our behavioral outcome of interest was the margin of stability (MoS) at the instant of foot strike, as an instantaneous measure of postural stability. The MoS is an established measure of dynamic stability that takes into account both the position and the velocity of the center of mass (CoM) relative to the boundary of the base of support (BoS). The MoS (in meters) is calculated according to Eq. (1).

1 $$ MoS = XcoM - BoS $$

2 $$ XcoM = x + \frac{ {\dot{{\text{x}}}} }{{\omega_{0} }} $$

with the extrapolated center of mass (XcoM, Eq. 2)^16^ derived from the CoM position *x* and its velocity $\dot{x}$ in the anterior–posterior direction, normalized by $\omega_{0}$ (the ratio between Earth’s gravitational constant *g* = 9.81 m/s^2^ and leg length *l* in meters, Eq. 3).

3 $$ \omega_{0} = \sqrt{\frac{g}{l}}  $$

A MoS well above zero indicates a stable posture. Yet, once some (theoretical) critical value of ‘good stability’ has been reached, any further increment in MoS is considered to not have additional relevance for maintaining dynamic stability. A low MoS indicates a less stable postural state as the CoM is approaching the boundary of the BoS. A MoS value of zero or lower means there is no more margin for the XcoM to the BoS and a fall is imminent if no corrective response is executed.

In forward trials, we used the toe marker of the stepping leg as the BoS. For backward foot strikes we used the heel marker of the stepping leg as the BoS.

#### EEG pre-processing

EEG and EOG data were preprocessed in MATLAB using functions of the EEGLAB toolbox^17^. Data were bandpass filtered (2–200 Hz, consecutive high-pass and low-pass 5th order Butterworth IIR filters, zero-phase shift). Channels were checked for flat lines, outliers (amplitude and kurtosis > 5 SD) and low correlation with other channels (threshold 0.7). After removing noisy channels, the average reference was computed, and the power at 50 Hz is estimated and channels or trials above 4 SD are rejected. Continuous data were downsampled to 512 Hz and epoched per trial with 3 s prior to and 6 s post perturbation onset. Noisy epochs were automatically rejected (amplitude threshold > 6 SD) before independent components analysis (Infomax ICA) was computed. Independent components were rejected based on being excessively noisy and of non-brain origin (mean = 10 accepted components, SD = 2.75 accepted components). Artifact-reduced EEG was obtained by back-projection of the retained independent components.

#### Generalized eigendecomposition

We applied generalized eigendecomposition (GED), a multivariate source-separation method, on the clean EEG data in order to derive a spatial filter that is optimized for theta (3–8 Hz) activity^18^. We performed GED to create a spatio-temporal filter optimized for theta activity, which is superior to ICA for hypothesis-driven dimension-reduction^18^. Computation of the GED on fewer components resulting from ICA cleaning does not affect the GED as GED is defined for any rank matrix^18^. Two covariance matrices were constructed corresponding to theta-band filtered data (matrix S) and broadband data (matrix R) (Eq. 4). The generalized eigendecomposition on these two matrices returns a set of eigenvectors (W) and corresponding eigenvalues ($\Lambda$), where the eigenvector associated with the largest eigenvalue is a set of channel weights (spatial filter) that maximizes the relative energy in the theta band.

4 $$ SW = RW\Lambda $$

This generalized eigenvalue equation solves the generalized Rayleigh quotient and is often used in machine learning and brain-computer-interface research^18–20^.

One spatial filter with a midfrontal topography and the largest eigenvalue, was selected per participant for further analysis of the EEG data. The GED component time series was computed as w^T^X, where X is the channel time series data. The corresponding spatial map was computed as w^T^S^20^.

#### Time–frequency analysis

The GED component time-series were further characterized through time–frequency decomposition. This was implemented by narrow-band filtering the time series at a range of frequencies through trial-by-trial convolution with complex Morlet wavelets. Equations (5) and (6) show the construction of the complex Morlet wavelets.

5 $$ \Psi_{f} = e^{i2\pi ft} e^{{ - t^{2} /2s^{2} }} $$

6 $$ s = \frac{n}{2\pi f} $$

where *t* represents time, *f* is frequency, *s* is width of the Gaussian modulating the complex sine wave and *n* stands for the number of wavelet cycles. The number of cycles per wavelet controls the temporal and spectral precision tradeoff. We used 40 frequencies logarithmically spaced between 2 and 60 Hz. Wavelet widths were logarithmically spaced from 4 to 12 cycles. We selected a condition-specific baseline window of − 1.4 to − 0.7 s relative to perturbation onset. Individual time–frequency data were averaged in the frequencies of the theta rhythm (3–8 Hz) per direction.

### Statistical analysis

To evaluate theta power increases after foot strike compared to baseline, sample-wise *t* tests of theta power were computed for each time point over 0–550 ms relative to foot strike to determine a difference relative to baseline theta power. This temporal window was selected based on the average time from foot strike to the end of platform motion (547 ms, that we rounded up to 550 ms for simplicity). The average baseline theta was averaged over a predefined window of − 1400 to − 700 ms relative to perturbation onset. To correct for multiple tests, *p*-values were corrected for false discovery rate (FDR)^21^ with statistical significance assessed for critical α < 0.05.

To evaluate directional differences in foot strike event latencies, we compared these latencies between backward and forward steps using *t* tests. In addition, to identify directional differences in theta dynamics time-locked to foot strike, we conducted sample-wise *t* tests over 0–550 ms. Given the multiple tests, *p*-values were corrected for FDR with statistical significance assessed for critical α < 0.05.

To determine the relation between theta and dynamic stability at foot strike, we computed Spearman correlation coefficients between the margin of stability at foot strike (averaged across trials for each participant and direction) and theta power at each time point between 0 and 550 ms relative to foot strike.

### Ethics approval and consent to participate

Participants provided written informed consent before participating in the experiment according to the experimental procedures approved by the Research Ethics Committee of the Radboud University Medical Center (Nijmegen, The Netherlands; Dossier NL67690.091.18). The experiments were conducted in line with the Declaration of Helsinki.

## Results

### Foot strike latency

From a total of 1203 collected trials (in the forward and backward direction) (mean = 80, SD = 1.7 trials per participant), 0.25% were rejected based on the stepping latency laying outside of the temporal window of constant velocity of the platform. An additional 1% of the trials were rejected due to missing marker data in the critical foot strike time window (see “Methods” section for rejection criteria).

**Figure 2:**
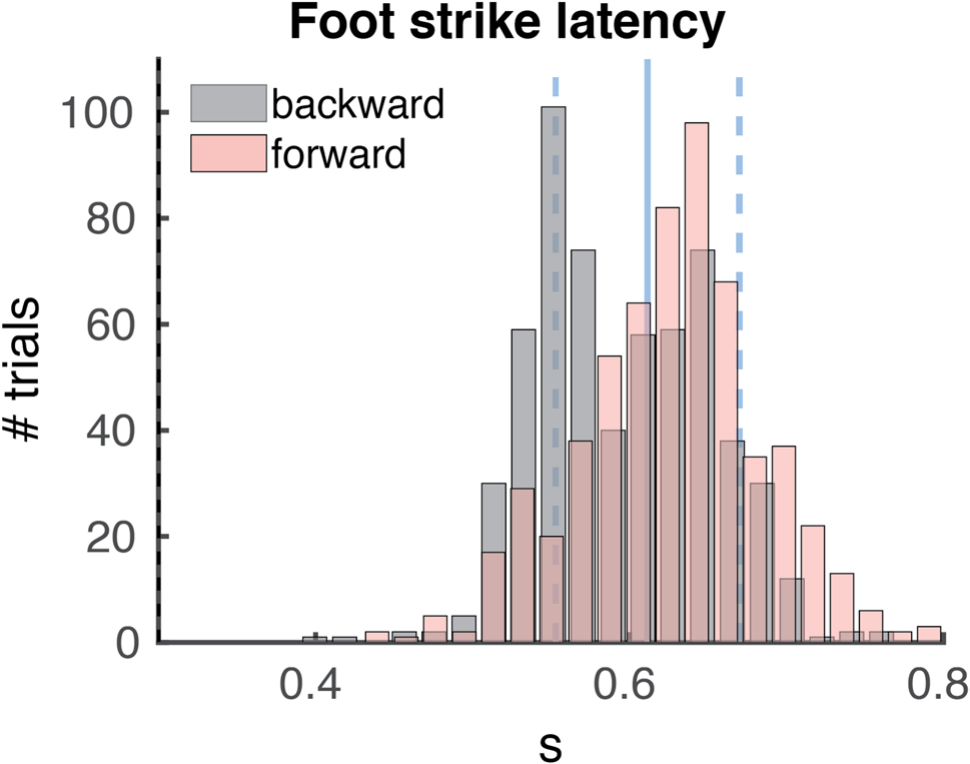
Foot strike latencies. Single trial foot strike latencies in the forward (red) and backward (grey) direction. The solid vertical blue line represents the overall mean foot strike latency (611 ms) and the dashed blue lines represent the standard deviation (58 ms). Presented foot strikes all occurred during the constant velocity phase of the platform perturbation (0.3–0.8 s).

Figure 2 shows the histograms of single trial foot strike latency distribution in the forward and backward direction. Across the forward and backward direction, we found an average foot strike latency of 611 ms (SD 58 ms) relative to perturbation onset. Foot strike latencies significantly differed between directions (t(14) = 3.09, p < 0.01) with faster foot strikes in the backward direction (mean = 596 ms, SD = 44 ms) compared to the forward direction (mean = 627 ms, SD = 46 ms).

### EEG

**Figure 3:**
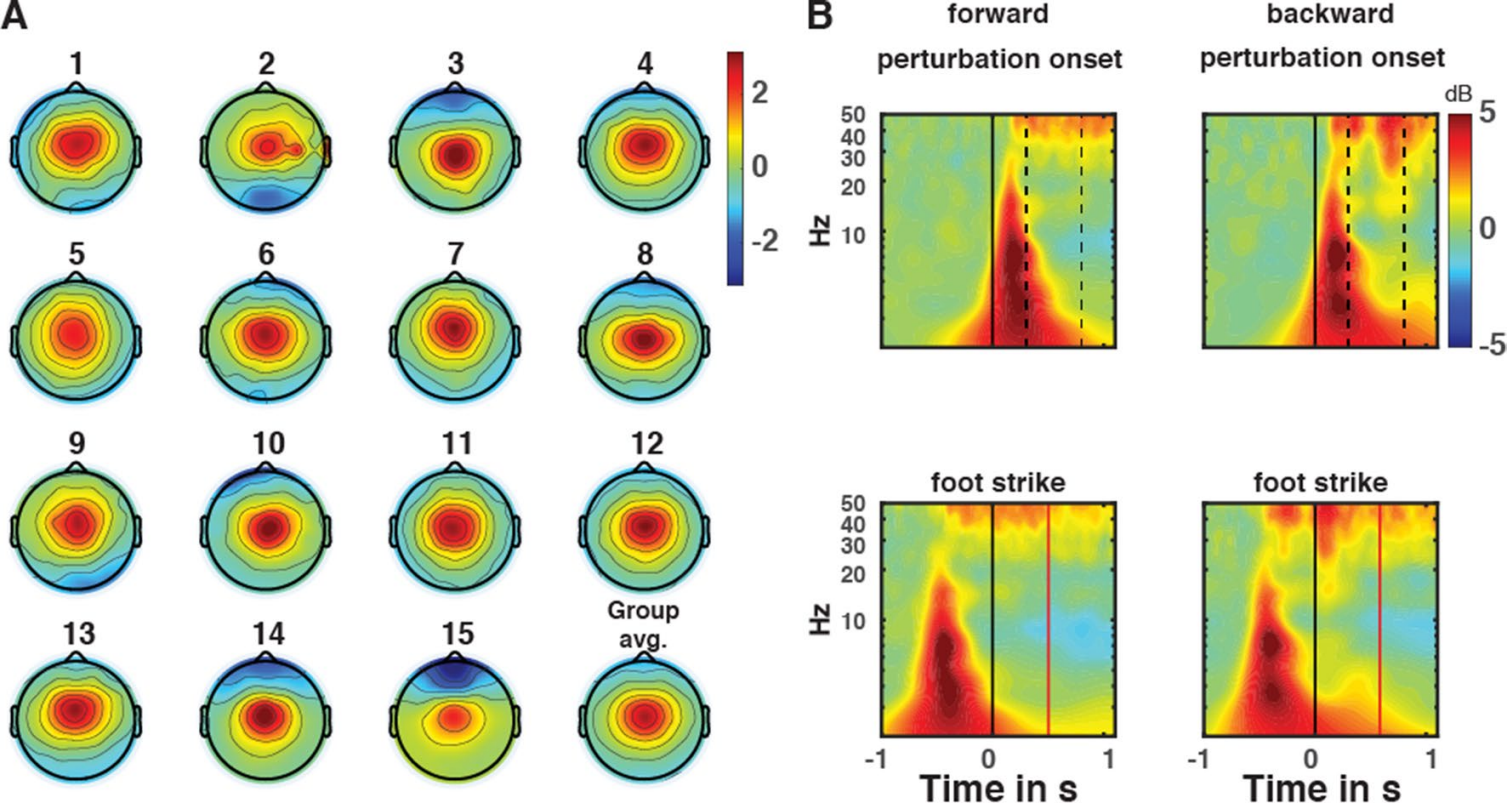
Characteristics of midfrontal GED component data. (**A**) Participant-specific scalp topographies of the spatial filter with the largest eigenvalue and a midfrontal topography (normalized scale). The group average (Group avg.) scalp topography is presented in the bottom right figure. (**B**) Group averaged time–frequency results. Top row illustrates forward and backward time–frequency analysis of perturbation time locked EEG data, with the vertical solid line indicating perturbation onset. The vertical dashed lines indicate the start of platform constant velocity (0.3 s) and the end of the constant velocity phase (0.8 s). Bottom row illustrates time–frequency analysis of foot strike event time locked EEG data. The black vertical line at 0 s indicates the moment of foot strike and the vertical red line represents the average end of platform motion relative to foot strike in the forward or backward direction. Figures were created with the EEGlab toolbox.

The GED analysis yielded midfrontal topographies for all participants (Fig. 3A). Time–frequency analysis of midfrontal GED component data time-locked to perturbation onset showed the characteristic patterns of midfrontal theta frequency power increase compared to baseline for both forward and backward directions (top panels Fig. 3B). In addition to theta, delta (0.5–4 Hz), alpha (9–12 Hz) and low-gamma (40–50 Hz) band power increase is observed following perturbation onset. Time locked to foot strike we observe a theta power increase (bottom panels of Fig. 3B). Note that because of our focus on balance monitoring and a priori hypothesis, we here restrict our analyses to the theta band. In the backward direction, we also observed a longer-lasting theta power increase that was not evident in the forward direction.

### Theta power following foot strike event

**Figure 4:**
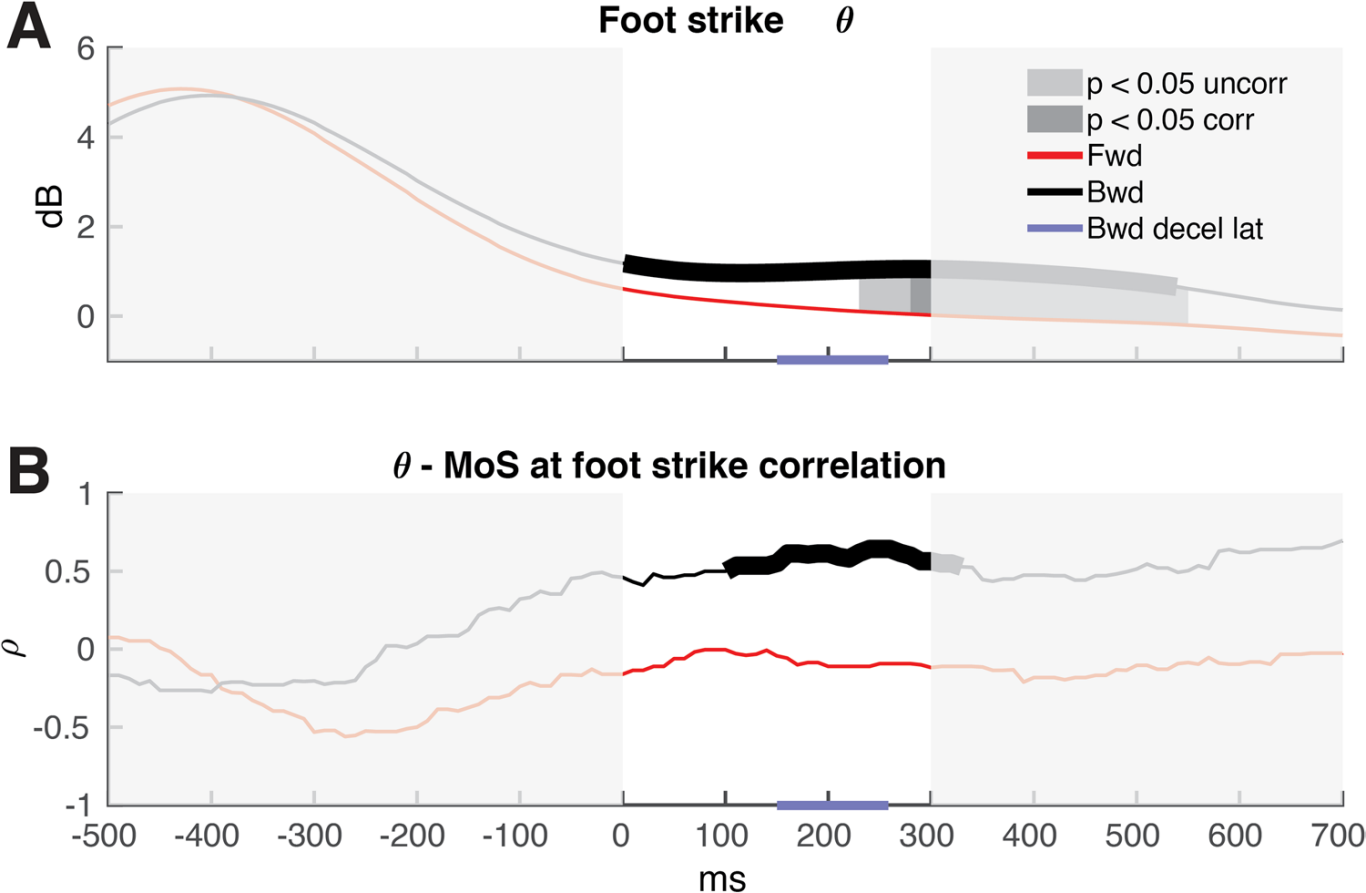
Foot strike time-locked theta time series. (**A**) Average theta time series for both stepping directions, with forward (Fwd) stepping indicated in red and backward (Bwd) stepping indicated in black. The highlighted white background indicates the temporal window where platform deceleration effects on theta dynamics are minimal. Significant difference in theta power between backward foot strikes relative to baseline with corrected false detection rate (fdr) is presented with a thicker line. Significant differences in theta power dynamics between stepping directions are indicated with dark grey (fdr corrected, ‘corr’) and light grey (uncorrected, ‘uncorr’). The horizontal blue line on the x-axis represents the average latency of platform deceleration onset (Bwd decel lat) relative to the instant of foot strike and standard deviation during backward stepping trials. (**B**) Spearman correlation over time of margin of stability (MoS) at foot strike with theta power. Significant correlation time window for backward foot strikes is indicated with a thicker line.

Average theta power during backward stepping trials was significantly stronger compared to baseline over 0–540 ms post foot strike (see thick black line Fig. 4A). Theta power following foot strike over 0–540 ms was Mean_forward_ = 0.1 dB, std_forward_ = 1.36 dB, mean_backward_ = 0.97 dB, std_backward_ = 1.39 dB. Forward stepping trials did not show differences in theta dynamics relative to baseline. In addition, we found significantly stronger foot-strike related theta power for backward compared to forward stepping over 280–550 ms post foot strike (see Fig. 4).

### Time series correlation between theta power and margin of stability

The average theta power in the backward direction per participant showed a significant positive correlation with the average MoS at foot strike (see Fig. 4B). This indicates that stronger theta power was observed in individuals with comparatively large margins of stability at foot strike. These correlations were significant between 110 to 340 ms following foot strike. We did not find a significant correlation for the MoS at foot strike and theta power in the forward stepping direction.

### Foot strike time locked theta dynamics may facilitate performance adaptation

**Figure 5:**
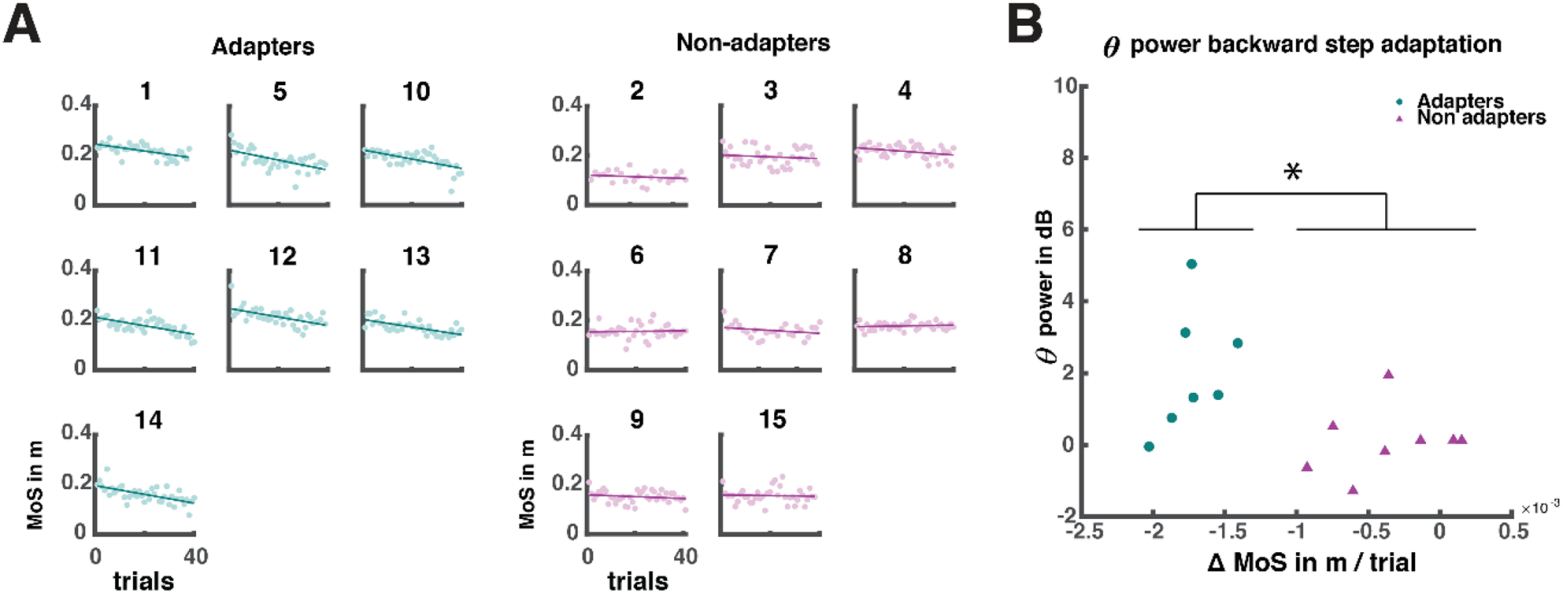
Adaptation of margin of stability at foot strike over the experimental time course for backward perturbations. (**A**) Participant specific margins of stability (MoS) are presented on the y-axis in meters and the trial order is presented on the x-axis. Adapters and non-adapters were classified based on significant correlations of MoS (in meters) change over trials. Numbers above plots represent the participant number. (**B**) Theta power as a function of adaptation slope in change of MoS in meters per trial. Significant difference in average theta power between adapters and non-adapters is indicated with *, p = 0.02.

We had expected to find *negative* correlations between the MoS at foot strike and theta power, as our previous study demonstrated that theta dynamics scale with the intensity of a balance perturbation (i.e. platform acceleration) as well as with its anticipated impact on postural stability^7,8^, suggesting that theta dynamics play a role in balance monitoring. However, contrary to our hypothesis and previous findings, we found a significant *positive* correlation between MoS and theta power in the backward stepping direction. To further investigate this unexpected finding, we performed an explorative post hoc analysis to evaluate the potential role of the observed midfrontal theta dynamics at foot strike. Importantly, the observed MoS values at foot strike were all well above zero (see Fig. 5A), suggesting that stability was not threatened at foot strike. Therefore, we considered other cognitive roles of foot strike related theta dynamics that may potentially explain our findings.

Theta dynamics have commonly been studied in simplified cognitive control and feedback paradigms (button press response performance), indicating that theta dynamics facilitate feedback processing and adaption of response performance during learning^22–24^. The positive correlation between theta and MoS may indicate that participants who initially take too large steps (as a function of greater MoS values) adjust their step lengths towards the end of the experiment in a feedback-based optimization process (indicated with high theta values). Thus, we investigated whether we could identify any participants who adapted their MoS at foot strike (that is, they saved energy by making smaller steps to successfully recover balance), with repeated perturbations and whether there would be a relation with theta dynamics. In particular, we investigated whether stronger theta power would be present in participants who adapted their stepping performance, thus gradually changing their MoS values at foot strike across repeated perturbations of equal and predictable intensity in the backward direction. Although generating smaller MoS may imply bringing balance at risk, MoS values were always large enough to achieve stability and thus may suggest that smaller MoS was generated to save energy rather than generating greater MoS. We labeled participants as “adapters” if MoS at foot strike decreased over trials (that is, they used smaller steps to successfully recover balance), or as “non-adapters” if their MoS was not significantly different over the course of the experiment (Fig. 5A). There were 7 adapters that individually showed significant and moderately strong negative correlations between MoS at foot strike and trial progression (⍴ = [− 0.66: − 0.55], p < 0.01), and 8 non-adapters with non-significant correlations between MoS and trial number (⍴ = [− 0.29: 0.1], all p > 0.05). Of note, the segregation of adapters and non-adapters through significant correlations, resulted in a median split based on correlation magnitude as observed in Fig. 5B.

We averaged the MoS over the first five trials to investigate whether the MoS in the beginning differed between adapters and non-adapters. Similarly, we checked whether the MoS at foot strike differed between the two groups over average of the last five trials. We found that over the first five trials, adapters generated greater MoS compared to non-adapters (p = 0.01, ranksum 43, median_adapters_ = 0.22 m, IQR_adapters_ = 0.03 m, median_non-adapters_ = 0.17 m, IQR_non-adapters_ = 0.04 m). MoS values averaged over the last five trials did not differ between adapters and non-adapters (p = 0.7, ranksum 45, median_adapters_ = 0.16 m, IQR_adapters_ = 0.05 m, median_non-adapters_ = 0.16 m, IQR_non-adapters_ = 0.06 m). In addition, foot strike MoS values averaged over the first five trials negatively correlated with the slope of adaptation (⍴ = − 0.58, p = 0.03).

For each participant, we averaged the theta power time series that was also used for the time series in Fig. 4. The start of the window was chosen to match the earliest time point where the correlation of MoS at foot strike with theta power reached significance, and the end of the window corresponded to ~ 900 ms post perturbation. As evident changes in theta dynamics are observed as early as 100 ms post event onset^5,7,25^, this time window limits the influence of possible theta dynamics induced by the platform deceleration (800 ms after perturbation onset; see “Methods”).

We observed stronger theta modulations at foot strike in adapters compared to non-adapters (Wilcoxon rank sum test median_*adapters*_ = 1.40 dB, IQR_adapters_ = 2.16 dB, median_non-adapters_ = 0.12 dB, IQR_non-adapters_ = 0.73 dB, ranksum = 44, p = 0.02), indicating that participants who adapted their MoS over the time course of the experiment showed stronger theta power following foot strike. The correlation of theta with adaptation slope (the change in MoS with trial count) did not reach significance (Spearman ⍴ = − 0.43, p = 0.11; Pearson r = − 0.48, p = 0.07) see Fig. 5B).

## Discussion

The goal of our study was to clarify the balance monitoring role of the theta dynamics following foot landing of a reactive stepping response. We expected that the foot strike event requires a cognitive balance monitoring assessment, which would manifest as an increase of midfrontal theta power. Our results indicate that midfrontal theta power increased following a foot strike for the backward direction only. In addition, we hypothesized that these theta dynamics represent the balance monitoring of the new postural state after foot strike and predicted that stronger theta dynamics scale with postural threat (i.e. lower MoS) at foot strike. Interestingly and opposite to our hypothesis, the correlation analysis of theta power with the MoS at foot strike revealed *greater* stability related to stronger theta power following foot strike, which contradicts previous findings of midfrontal theta and its relation to postural stability. In a post hoc analysis we explored whether midfrontal theta dynamics related to response adaptation by distinguishing between individuals who adapted their MoS during stepping responses over the experimental duration and those who did not (i.e., adapters vs non-adapters). The results of this analysis showed that cortical theta power of adapters was significantly stronger post foot strike compared to non-adapters.

### Cortical theta dynamics following foot strike

The finding of a significantly increased theta power following foot strike of a reactive backward step may seem in agreement with its proposed role in stability monitoring, yet the observed positive correlation of theta power with MoS argues against such a role in this phase of the balance recovery response. For the interpretation of balance monitoring related cortical dynamics, it is important to note that the increased theta power reported here already presented itself in the constant velocity phase of the perturbation profile, thus excluding a potential confounding effect of platform deceleration on theta power, at least for the initial ~ 300 ms post foot strike.

The presently observed positive correlation between MoS and theta power is inconsistent with previous literature that reported negative correlations between theta power and postural stability, suggesting that theta indexes to what extent stability is at risk^5,26^. It must be mentioned, though, that in our experiment the participants’ stability was never at risk once the stepping foot had landed, as the observed MoS values were well above zero (~ 17.5 cm on average) and participants never needed a second step to recover balance. This lack of challenge to stability following foot strike in response to our relatively low-intensity perturbations may (at least partly) explain the discrepancies with previous findings. Yet, the significant *positive* correlation that we found raised the question whether the theta dynamics following foot strike may have facilitated a different cognitive process. This led us to conduct a secondary explorative analysis, the results of which will be elaborated on below.

### Foot strike direction and theta

Our results indicate differences in cortical theta dynamics for foot strike directionality with greater theta power dynamics after backward compared to forward steps. It is interesting that we only observed a significant increase of midfrontal theta in the backward direction, which suggests that there may be a greater need for cortical involvement following foot strike in this direction. Despite the fact that the perturbation intensity was not very challenging in either direction, backward reactive steps may still have been perceived as more difficult than forward reactive steps, with MoS values in the latter direction being larger by ~ 4 cm on average. While the imposed perturbation intensities in this study were higher than the stepping thresholds in either direction, stepping thresholds also differ across direction, with lower thresholds in the backward (0.66 m/s^2^) compared to forward direction (1.09 m/s^2^) for the presently used perturbation waveform^13^. Yet, the perturbation intensities where young participants need more than one step to recover balance are substantially higher than those used in the present study (1.5 m/s^2^), as previous work has reported multiple stepping thresholds of 4.5 m/s^2^ (forward) and 3.5 m/s^2^ backward^27,28^. Hence, while in the present study stability was never at risk following foot strike, the relative challenge was slightly larger in the backward than the forward direction.

Another possible explanation for the directional differences may be sought in the role of visual input that differs between stepping directions. Theta dynamics have been shown to increase in walking with eyes closed compared to eyes open^29^, suggesting that differences in theta dynamics arise from cortical engagement of sensory areas when visual input is present to a lower extent or missing at all. During forward stepping we can rely on visual input of where we will place our foot while stepping, whereas in the backward stepping direction this information is not available to us. Therefore, the lack of visual input in the backward direction may result in an increase of theta dynamics during backward stepping.

### Theta power time locked to foot strike may facilitate monitoring of step performance

Cognitive control and feedback processes are facilitated by the theta frequency band, suggesting that theta dynamics are involved in feedback processing and adaptation of response performance during task learning^22,23,30^. Therefore, we explored whether a similar process may have been at work in our experiment by identifying whether theta dynamics differed between participants who did and those who did not adapt their postural stability at foot strike over the time course of the experiment.

We reasoned that naïve participants would gradually become more familiar with the platform perturbations—which intensities were kept constant throughout the experiment—and learn to optimize their step responses. Successful stepping with incrementally smaller MoS (i.e. taking smaller steps) may have reflected learning throughout the course of the experiment that enabled participants to maintain stability while adopting a more energetically favorable strategy. We did not instruct participants to adapt their behavior, and thus the adaptation effects reflected a spontaneous strategy that approximately half of the participants deployed.

We expected this gradual step adaptation to be most evident in those participants who took (unnecessary) large steps at the start of the experiment with, consequently, a higher *average* MoS value across the experiment. We reasoned that this may explain the unanticipated *positive* correlation between theta power and MoS in our experiment. The explorative post hoc analysis indeed revealed that adapters showed stronger theta modulations following foot strike than did non-adapters. These findings are in line with previous studies reporting stronger theta modulations in participants who adapted their task performance in other types of tasks^22–24^. In addition, adapters showed greater theta dynamics compared to weak adapters in a performance feedback task^31^. Our present results may therefore hint towards a role of midfrontal theta power representing performance monitoring post foot strike for optimizing the reactive stepping response, rather than monitoring whether balance is at risk, at least for the particular perturbation protocol that we used. Future studies may further address this by systematically investigating trial to trial changes in theta dynamics and adaptation of step performance in response to balance perturbations to investigate whether the correlation between MoS and theta changes over time as a function of adaptation.

### Limitations

We identified some limitations that should be considered for future studies investigating cortical dynamics time locked to reactive balance steps. Importantly, we identified that theta dynamics may facilitate different monitoring roles throughout the postural response and therefore the experimental design should be carefully considered with regard of the specific role of interest.

Perturbations used in our experimental paradigm may not have been challenging enough to study the monitoring of postural stability at foot strike in healthy young participants. In our study, we used fixed intensities that were well below their multiple stepping threshold in both directions. Yet, it must be mentioned that the presently used experimental protocol was devised for comparing reactive stepping behavior across multiple groups, including people with stroke. Because people with stroke experience substantial difficulties in reactive stepping, we purposely selected a relatively low perturbation intensity of 1.5 m/s^2^, which roughly corresponds to the 25% percentile of multiple stepping thresholds in people with stroke as reported by^27^. It would therefore be of interest to evaluate whether the anticipated negative correlation between theta dynamics and MoS may be present in people with stroke or other clinical populations with impaired reactive stepping responses. Yet, for future studies in young adults we suggest to use higher perturbation intensities that challenge the participants’ postural stability at foot strike.

In addition, balance perturbations of similar intensities are experienced as more difficult in the backward than the forward direction. Thus, our results may be biased to finding stronger theta dynamics in the backward direction. Therefore, we suggest future studies to use equally challenging perturbations in either direction, for instance by tailoring these to the individual’s direction-specific multiple stepping threshold, to allow better comparison of cortical markers.

While it would have been of interest to study trial-to-trial MoS adaptions, the randomized perturbation directions only allowed us to focus on long term adaptation over the course of the experiment. Future studies on trial-to-trial adaptations should include sequences of multiple perturbations in the same direction. In addition, such studies may want to use shorter ITI durations than those used in the present study (8 s), as it was previously shown that a potential learning effect in a Stroop task paradigm, was reduced when the inter-trial interval (ITI) was greater than 2 s^32^. Yet, it remains to be studied whether a similar decay is also applicable to short-term adaptations in balance responses.

### Clinical implications

Many neurological conditions cause balance impairments that drastically reduce the quality of life of people affected. In addition to the aforementioned studies of reactive stepping related cortical dynamics in people with stroke, our present results may also inform future studies in people with Parkinson’s disease. It is known that the dopaminergic pathway, involving the striatum and the rostral cingulate zone are affected in Parkinson’s disease^33^. These brain areas are also involved in learning and action adaptation from response outcomes, through theta dynamics (and the event related negativity, ERN/ N1) representing reinforcement-learning to optimize task performance (For an extensive review on performance monitoring, see^34^. Therefore, clarification of foot strike related cognitive underpinnings are an interesting area of study for gaining novel insight into mechanistic underpinnings of balance control deficits in people with PD.

## Conclusion

We aimed to investigate the balance monitoring role of midfrontal theta dynamics following foot strike during reactive stepping. The positive correlation between theta dynamics following backward foot strikes and postural stability contradicts previous literature on the relation of theta dynamics and stability. Based on our post hoc analysis results we speculate that the observed increase of theta power following foot strike was not related to stability monitoring but instead may indicate cortical dynamics related to performance monitoring of the response outcome. It is an interesting question for future studies whether theta dynamics following foot strike may reflect the anticipated balance monitoring role by studying young adults at higher perturbation intensities, or balance-impaired individuals at the presently used intensities. When selecting the experimental protocol, these future studies should carefully consider the possible cognitive roles theta may facilitate at foot strike.

## Data Availability

The datasets generated and/or analyzed during the current study are not publicly available due to ongoing data collection and analysis for the “*Roads to recovery*” study. Data from this study are available from the corresponding author on reasonable request.

## Abbreviations

CoM: Center of mass
BoS: Boundary of the base of support
MoS: Margin of stability
xCoM: Extrapolated center of mass
EEG: Electroencephalogram
EOG: Electrooculogram
ERN: Event related negativity
ICA: Independent component analysis
GED: Generalized eigendecomposition
FDR: False discovery rate
PD: Parkinson’s disease
